# Supplementary material for: Laboratory Evaluation of a Wearable Instrumented Headband for Rotational Head Kinematics Measurement
Source: Ann Biomed Eng. 2025 May 16;53(8):1944–61. doi: 10.1007/s10439-025-03746-7 (PMC12283827; doi:10.1007/s10439-025-03746-7)
Supplement: Supplementary file 1 — (pdf 1504 KB) [file 10439_2025_3746_MOESM1_ESM.pdf]

Supplementary Materials: Laboratory evaluation of a wearable instrumented headband for rotational head kinematics measurement

Journal: Annals of Biomedical Engineering

Anu Tripathi[1], Yang Wan[2], Sushant Malave[3], Sheila Turcsanyi[4], Alice Lux Fawzi[5], Alison Brooks[6], Haneesh Kesari[2], Traci Snedden[7], Peter Ferrazzano[4], Christian Franck[5], Rika Carlsen[1]*

[1]Department of Engineering, Robert Morris University, Moon Township, PA,USA.
[2]School of Engineering, Brown University, Providence, RI, USA.
[3]Team Wendy, Cleveland, OH, USA.
[4]Waisman Center, University of Wisconsin–Madison, Madison, WI, USA.
[5]Department of Mechanical Engineering, University of Wisconsin–Madison, Madison, WI, USA.
[6]Department of Orthopedics and Rehabilitation, University of Wisconsin–Madison, Madison, WI, USA.
[7]School of Nursing, University of Wisconsin–Madison, Madison, WI, USA.

*Corresponding author email: carlsen@rmu.edu;
Contributing author email: tripathia@rmu.edu; yang_wan@brown.edu; smalave9@gmail.com; saturcsanyi@wisc.edu; afawzi@wisc.edu; brooks@ortho.wisc.edu; haneesh_kesari@brown.edu; tsnedden@wisc.edu; ferrazzano@pediatrics.wisc.edu; cfranck@wisc.edu;

1. CHAMP Checklist

No.	Checklist Item	Current Study Specifications	Reported on Page No
1.	**Sensor Technology and Specifications**		
	a. Device model name	a. 5 Blue Trident sensors embedded in Storelli headband	Pg. 4
	b. Sensor type	b. The Blue Trident sensor has a triaxial gyroscope, a triaxial low-g accelerometer, and a triaxial high-g accelerometer	Pg. 4
	c. Sensor sample rate	c. Gyroscope – 1125Hz<br>Low-g accelerometer – 1125 Hz<br>High-g accelerometer – 1600 Hz	Pg. 4
	d. Sensor magnitude range	d. Gyroscope – ± 2000 deg/sec per axis<br>Low-g accelerometer – ± 16 g per axis<br>High-g accelerometer – ± 200 g per axis	Pg. 4
	e. Device hardware version numbers (If applicable)	e. Blue Trident App (Capture.U 1.4.0 Update 10 Mar 2022)	Pg. 4
	f. Recording trigger threshold	f. The sensors record data continuously after manually starting the recording on the Capture.U app at the beginning of the session (no trigger threshold)	Pg. 4
	g. Pre-trigger duration	g. Continuous data available from the beginning of the session	Pg. 4
	h. Post-trigger duration	h. Continuous data available until the end of the session, 1 GB data, or 12 hours, whichever is less	Pg. 4
	i. Device form factor and attachment	i. The sensors are embedded in slots carved out of the inner surface of the headband and attached using Velcro.	Pg. 4
2.	**Surrogate Selection**		
	a. Surrogate used	a. A Hybrid III (HIII) 50th percentile male (Humanetics Innovative Solutions, Inc.) head and neck were rigidly bolted to a flat elevated surface.	Pg. 4
	b. Inertial properties of surrogate	b. This ATD had the inertial properties of a 50th percentile male head.	Pg. 4
	c. If applicable, modifications made to standard surrogates	c. No modifications made	N/A
	d. If applicable, corresponding neckform and/or other body segments used	d. A standard H-III neck	Pg. 4
	e. Modifications made to standard neckforms, if applicable	e. No modifications made	N/A

	f. Validation of the surrogate<br>g. Mounting of the device on the surrogate<br>h. Factors related to coupling of the device to the surrogate	f. No modifications made<br>g. Size 6 storelli instrumented headband was mounted on the forehead of the H-III head.<br>h. Not studied	N/A<br>Pg. 4<br>N/A
**3.**	**Test Conditions**<br>a. Test device<br>b. Impactor surface and mass<br>c. Surrogate orientation and mounting, if applicable<br>d. Impact velocity<br>e. Impact duration<br>f. Impact location<br>g. Impact direction<br>h. Number of trials<br>i. If applicable, helmet manufacturer/ model name<br>j. Repeatability and reproducibility of test conditions	a. The ATD was impacted with a soccer ball launched from a JUGS machine (JUGS Sports, Inc.) located five meters away.<br>b. The soccer ball was Adidas size 5 (~70 cm circumference) with 12 psi air pressure.<br>c. The Hybrid-III head was oriented facing the JUGS machine for the frontal impacts and was rotated axially at an increment of 45° for the four successive impact locations (front-side, side, back-side, and back).<br>d. Soccer ball launch speed was 13 or 16 m/s.<br>e. The soccer ball impacts lasted approximately between 20-30 ms in contact with the head<br>f. Front, front-side, side, back-side, and back.<br>g. Soccer ball direction is kept the same while the head is rotated to change the impact location. The impact direction approximately passed through the headform's CG.<br>h. 20 trials at the front and front-side impact locations; 10 trials at the side, back-side, and back impact locations.<br>i. Not applicable<br>j. The test setup was designed to replicate field soccer headers using a JUGS machine. Different trials resulted in the peak rotational velocities (PRV), peak rotational accelerations (PRA), and peak linear accelerations (PLA) to range from 4.5 − 21 rad/s, 700 − 4766 rad/s^2, and 100 − 475 m/s^2 as measured by the reference DTS sensor.	Pg. 4<br>Pg. 4<br>Pg. 4<br>Pg. 4<br>Pg. 7<br>Pg. 4<br>Pg. 4<br>Pg. 4<br>N/A<br>Pg. 4
**4.**	**Reference Sensor Measurement**<br>a. Reference sensor type and model<br>b. Reference sensor mounting<br>c. Reference sensor sampling rate<br>d. Reference sensor magnitude range	a. DTS 6DX PRO-A reference sensors (Diversified Technical Systems, Inc.) with a triaxial accelerometer and triaxial gyroscope.<br>b. Rigidly bolted at the H-III headform center of gravity<br>c. Accelerometer – 10 kHz<br>Gyroscope – 2 kHz<br>d. Accelerometer – 2000 g<br>Gyroscope – 8000 deg/sec	Pg. 4<br>Pg. 4<br>Pg. 4<br>Pg. 4

	e. Reference sensor filtering<br>f. Time synching of reference sensor to head kinematic device	e. The DTS translational acceleration data was filtered using a low-pass Channel Filter Class (CFC) 1000 filter at 1650 Hz, and angular velocity data using CFC 180 at 300 Hz.<br>f. The headband and the DTS data were time-synced such that the first data point that crossed the 3 g threshold was set to time t = 0.	Pg. 8<br>Pg. 8
**5.**	**Advanced Post-Processing**<br>a. Data transformation<br>b. Kinematic data filtering<br>c. Other post-processing techniques<br>d. Event removal	a. Data from all headband sensors transformed to the same reference frame and then averaged<br>b. A novel adaptive wavelet-based filtering technique<br>c. N/A<br>d. No events were removed	Pg. 6<br>Pg. 7-9<br>N/A<br>N/A
**6.**	**Analytical Methods and Data Repo**<br>a. Validation metrics, including equations used to derive metrics, if applicable<br>b. Statistical and analytical methods for comparison	a. Time histories and peak of the rotational velocities and peak rotational accelerations were validated.<br>b. The time histories were evaluated using the Correlation and Analysis (CORA) score which provides the cross-correlation between the two curves based on the phase correlation, peak magnitudes, and shape of the curves. The peak values were evaluated using correlation coefficients, normalized root mean square error, concordance correlation coefficient, and Bland-Altman analyses.	Pg. 9<br>Pg. 9

2. Schematics showing the sources of noise in the headband data and steps to remove the noise

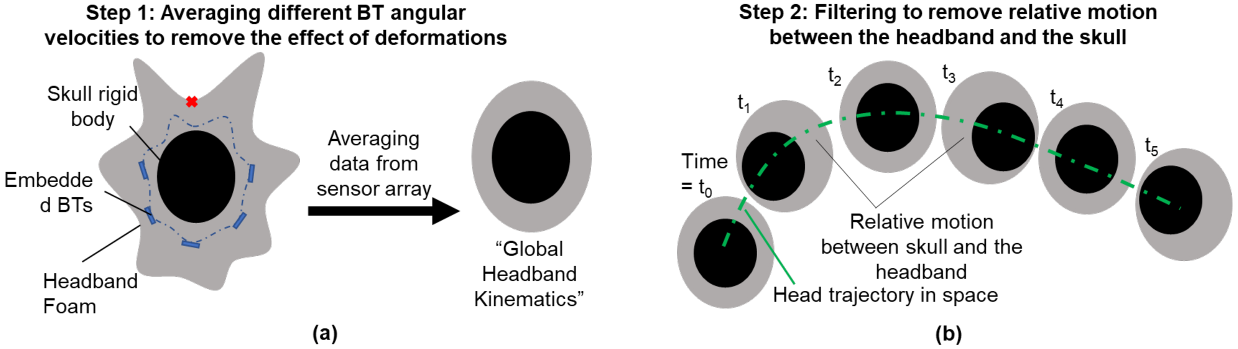

Figure S1 a) Schematic showing deformation in the headband and averaging of the angular velocity vectors from the sensor array to provide an estimate of the headband (HB) angular velocity (ω_h); b) Schematic showing relative motion between the head and the headband that should be removed to reconstruct the head kinematics (ω_{hf}).

3. Effect of relative angle between the impact and the sensor location on the angular velocity

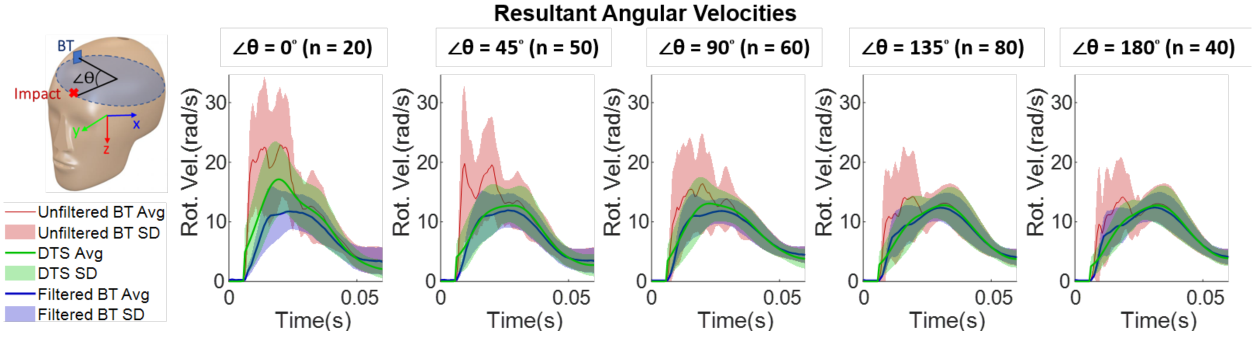

Figure S2 Average angular velocities and standard deviations from DTS (green), unfiltered BT (red), and filtered BT (blue) for all headers within each relative angle group 0, 45, 90, 135, and 180 (angle between the BT and impact locations). The plots show that as the relative angle increases, the noise in the unfiltered BT data decreases, and the match between the DTS and filtered BT improve.

4. Effect of the impact location on the noise and signal overlap

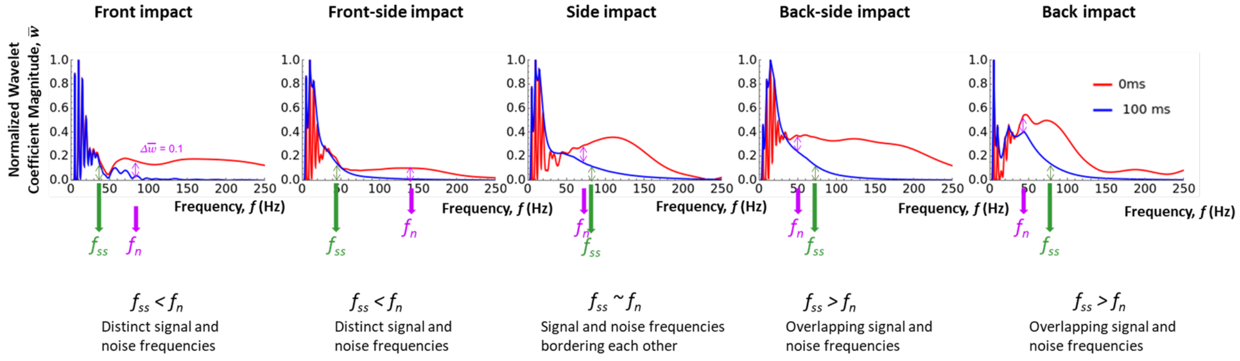

Figure S3 Normalized wavelet coefficients at the beginning (t = 0 ms, red) and at the end (t = 100 ms, blue) of the impact for a representative header at the front, front-side, side, back-side, and back locations. The plots show that as the impact moves to the back of the head, where the sensors are located, the frequency where noise starts (f_n) begins to become lower than where signal ends (f_{ss}), i. e. $f_{ss} > f_n$.This results in less efficient filtering of the impacts on the side, back-side, and back of the head.

5. Effect of the impact location on the peak rotational velocity and peak rotational accelerations

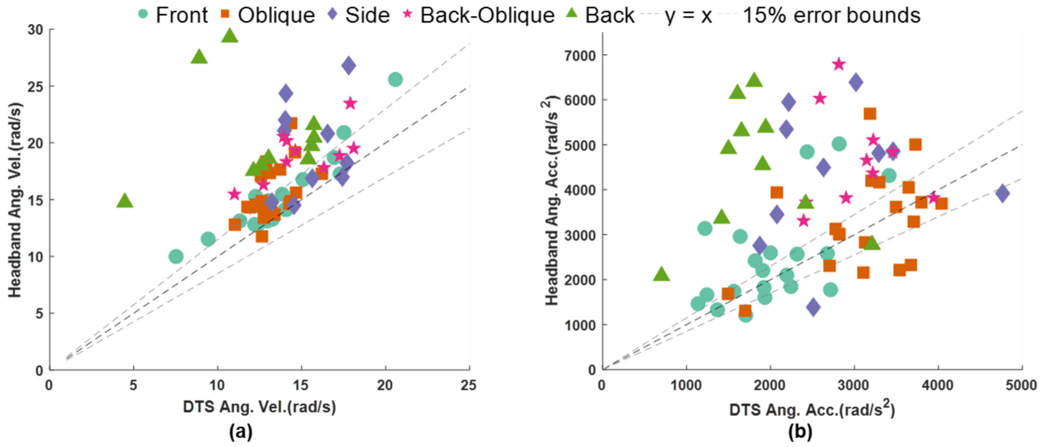

Figure S4 Linear regression plots comparing the (a) peak rotational velocity (PRV) and (b) peak rotational acceleration (PRA) measured from the instrumented headband and reference sensors for different impact locations.

6. Bland-Altman Analyses

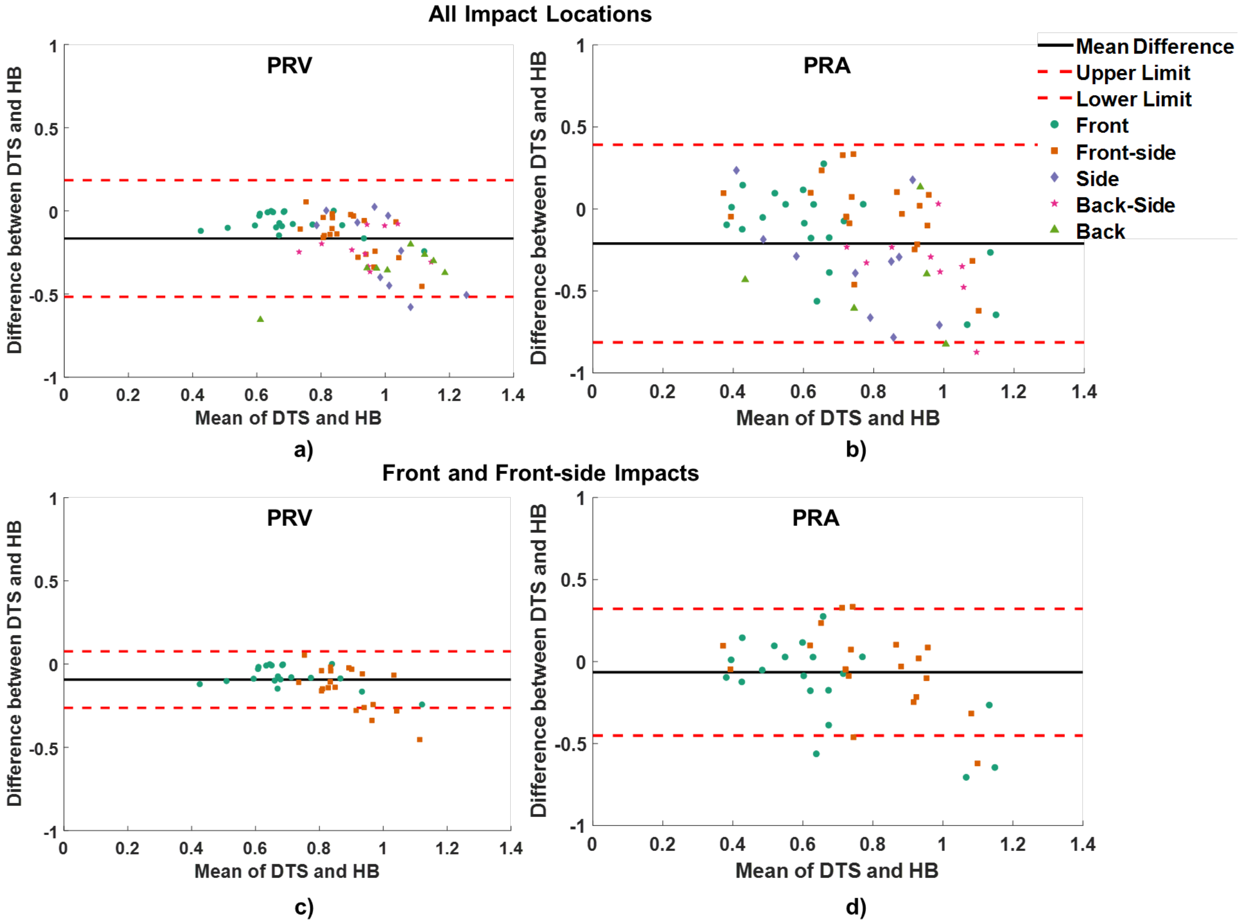

Figure S5 Bland-Altman plots showing the mean bias and limits of agreement between the headband and the reference sensors for a) PRV data from all impact locations, b) PRA data from all impact locations, c) PRV from impacts to the front of the head, and d) PRA from impacts to the front of the head, where PRV is the peak rotational velocity and PRA is the peak rotational acceleration.

7. Effect of filtering on statistical correlations with the reference peak kinematics

Table S2 The statistical correlation measures (r, CCC) and the normalized root mean square error (NRMSE) are shown for the peak rotational velocity (PRV) and peak rotational acceleration (PRA) based on the unfiltered back sensor data, unfiltered averaged data, filtered averaged data using a simple Butterworth filter with a constant cutoff frequency of 250 Hz [14], and filtered averaged data using an adaptive cutoff from the wavelet method. These statistical measures show significant improvements after the averaging step and after applying the adaptive filtering method for the front and front-side impacts, as well as for all impact locations. The results show that the adaptive filter outperforms a simpler filter with a single fixed cutoff frequency.

		PRV			PRA		
**Method**		**r**	**CCC**	**NRMSE**	**r**	**CCC**	**NRMSE**
**Unfiltered back sensor**	**All Impact Locations**	0.22	0.05	1.24	0.19	0.01	7.7
**Unfiltered averaged**		0.32	0.15	0.66	0.09	0.02	4.2
**Filtered average (Constant Cutoff) [14]**		0.34	0.17	0.63	0.12	0.02	2.70
**Filtered average (Adaptive Cutoff)**		0.42	0.25	0.50	0.34	0.33	0.41
**Unfiltered back sensor**	**Front and Front-side Impacts**	0.15	0.06	0.67	0.31	0.04	3.8
**Unfiltered averaged**		0.67	0.45	0.26	0.22	0.05	2.1
**Filtered average (Constant Cutoff) [14]**		0.70	0.49	0.26	0.23	0.07	1.50
**Filtered average (Adaptive Cutoff)**		0.80	0.62	0.20	0.63	0.55	0.28

8. Comparison of the current headband against existing instrumented mouthguards

Table S3 The correlation coefficient (*r*) for *all impact locations* from mouthguard studies show significantly higher correlations with the reference data than the instrumented headband from this study for both the peak rotational velocity (PRV) and the peak rotational acceleration (PRA). It should be noted that the mouthguards were tested using linear impactors for helmeted or unhelmeted conditions, and some studies only provided angular acceleration results. The study where the effect of unclenched jaws was incorporated showed lower *r* values for PRV and PRA.

Sensor	Impact Type	Helmet	Correlation Coefficient PRV	Correlation Coefficient PRA
Unfiltered back sensor (Current Study)	Soccer Ball	No	0.22	0.19
Unfiltered headband (Current Study)			0.32	0.09
Filtered headband (Current Study)			0.42	0.34
Prevent Boil and Bite MG (Keiffer et al. [14])	Linear Impactor	Yes	-	0.98
		No	-	0.95
Prevent Custom MG (Keiffer et al. [14])		Yes	-	0.97
		No	-	0.91
Vector MG (Keiffer et al. [14])		Yes	-	0.61
Wake Forest MG (Rich et al. [16])		No	>0.99	0.98
Wake Forest, Helmeted (Miller et al. [17])		Yes	0.99	0.91
			1	1
No mandible Helmeted (Kuo et al. [18])		Yes	0.99	0.95
Unclenched Mandible Helmeted (Kuo et al. [18])			0.84	0.40
Clenched Mandible Helmeted (Kuo et al. [18])			0.99	0.89

9. Effect of the soccer ball speed on the peak kinematics reconstruction

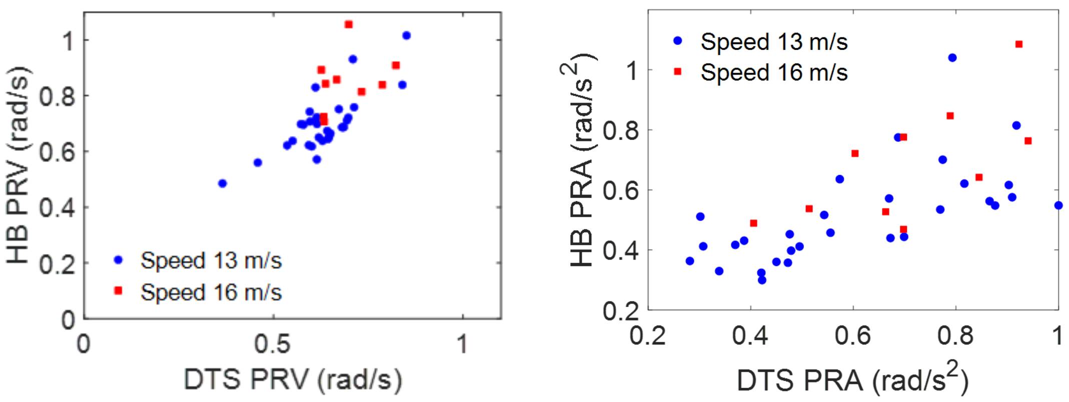

a) **b)**

Figure S6 Peak angular velocity (PRV) and peak angular acceleration (PRA) correlation between the headband (HB) reconstruction and the reference DTS data for two ball speeds: a) 13 m/s (blue) and b) 16 m/s (red), representative of the range of ball speeds measured in youth soccer. The results indicate that for the normal range of soccer ball speeds, the filtering method results in adequate head kinematics reconstruction.

10. Kinematics data from the BT directly affixed to the ATD surface using Velcro

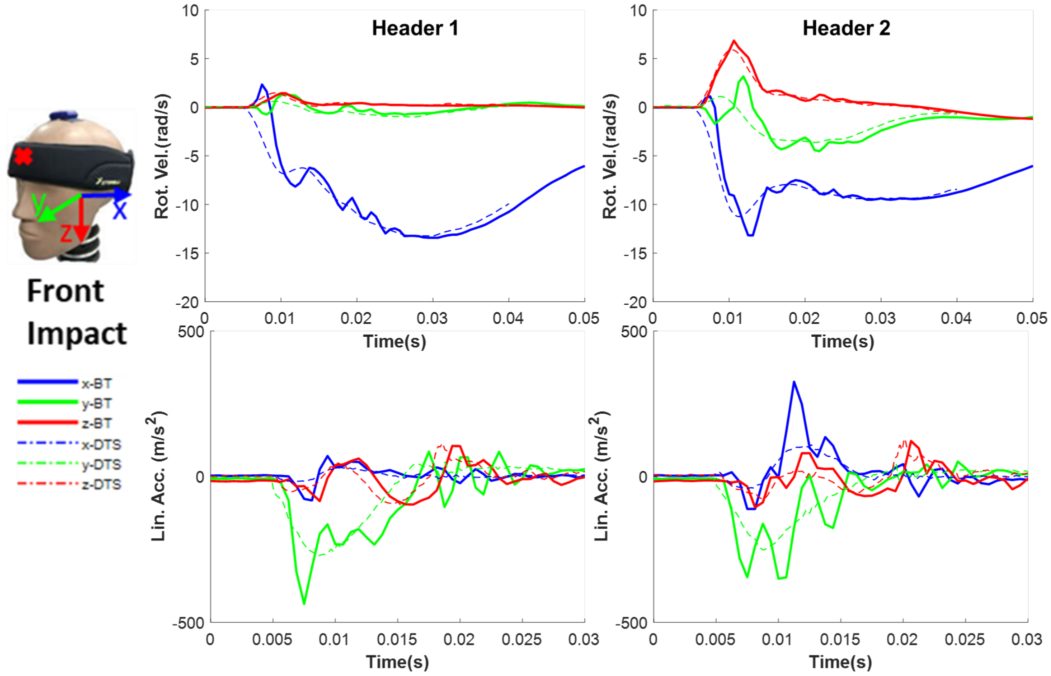

Figure S7 The crown BT (unfiltered) vs DTS (filtered) angular velocities and translational accelerations for two representative headers. These plots show lower noise in the signal when the BT is directly attached to the surface of the ATD using Velcro.
